# Sexual conflict underlying external female genital mutilation in spiders: assessing whether females benefit from multiple matings

**DOI:** 10.1098/rspb.2024.2722

**Published:** 2025-01-29

**Authors:** Kensuke Nakata, Yusuke Shigemiya

**Affiliations:** ^1^ Faculty of Contemporary Society, Kyoto Women’s University, Kyoto, Japan; ^2^ Faculty of Applied Information Technology, Nagasaki Institute of Applied Science, Nagasaki, Japan

**Keywords:** spider, reproductive output, sexual conflict, multiple matings, genital mutilation, paternity

## Abstract

External female genital mutilation (EFGM) is a type of traumatic mating in which males damage female genitalia, resulting in the loss of female re-mating ability. This study examined whether sexual conflict underlies EFGM by examining the possible female reproductive costs from the decreased number of matings in spider, *Cyclosa argenteoalba*. The female typically receives sperm from a male twice during a mating bout. We manipulated both the number of matings and the number of male partners of females and compared their reproductive outputs. The results indicated that females receiving sperm three times—equivalent to one and a half matings from two males—laid more egg sacs with more eggs per egg sac than control females that received sperm twice from one male. The control females laid more egg sacs and fewer eggs per egg sac than females that received sperm once. There was no significant difference in reproductive output between the control females and females that received sperm twice from the two males. These results indicate that females benefit from multiple matings but not from mating with multiple males, supporting the sexual conflict hypothesis. Our study has implications for our understanding of the evolution of harmful mating.

## 1. Background

In sexually reproducing organisms, the strategy for maximizing fitness often differs between males and females, resulting in sexual conflict [1]. Several male strategies have evolved to resolve sexual conflicts, leading to advantages for males. For example, in several mammals, infanticide by a newly established dominant male is expected to increase the number of his offspring, whereas females lose their offspring sired by a previous mating partner [2]. The ejaculate of *Drosophila* flies contains chemicals that reduce the longevity of females. After mating, females tend to stop searching for another male and begin laying eggs using the available sperm; that is, males manipulate females to ensure their offspring through chemicals in the ejaculate [3]. Sexual conflict is also an evolutionary driving force for female counter-adaptation. An example of this can be found in certain water striders. For female water striders, mating often incurs costs, whereas males try to mate with females repeatedly. In response, females equipped with structures in their abdomens can easily hinder male mating attempts [4].

External female genital mutilation (EFGM) is a strategy used by the males of several spider species to secure paternity [5,6] and it differs from male genital mutilation, which serves as a mating plug in other spiders [7]. In most spiders, both male and female genitalia exhibit complex shapes, and mechanical coupling is necessary for successful mating [8]. In species with EFGM, males damage the female genitalia, specifically by mutilating a scape, which is a small projection attached to the epigynum (external female genitalia). This scape is necessary for the male pedipalp (secondary genitalia) to grasp before palpal insertion [9]. Thus, genital coupling becomes impossible after mutilation; that is, mutilated females lose their re-mating ability. Mutilated females respond to courtship by subsequent males and attempt to mate, but these attempts are always unsuccessful. In a few species the mutilation success is high, resulting in functional monandry [5,6].

EFGM seems to be a male strategy that has evolved during sexual conflict, as observed in other strategies to secure paternity, such as mating plugs [10] or traumatic insemination [11]. Mutilator males benefit from securing paternity. However, whether mutilated females suffer any costs remains to be fully elucidated. Whether sexual conflict underlies EFGM remains an important question that must be answered, and this study examines this question.

One potential cost of EFGM is the physiological stress from injury and subsequent repair [12]. However, for *Larinia jeskovi*, an orb-web spider with EFGM, mutilation does not change the oxidation status, which is an indicator of physiological stress that is significantly affected by leg tip amputation [13]. Another supposed cost is the decreased reproductive output from the loss of the benefits of mating with multiple males [14]. This study examined whether females suffer the latter cost. Namely, we aimed to examine whether multiple matings provide benefits to females in terms of the production of viable eggs. These benefits may be genetic; that is, females benefit from choosing more appropriate sperm from males of different genotypes [15], or material, such as nuptial gifts and sperm replenishment from increased sperm transfer [16,17]; alternatively, male ejaculation may trigger increased egg production. Genetic benefits arise from an increased number of mating partners, while material benefits can be realized through an increased mating frequency and not necessarily an increased number of mating partners. Experiments that manipulate the number of matings and partner males and examine the effect of manipulation on female reproduction—the number of eggs laid or hatchling success—are essential to elucidate whether female spiders suffer from forced monandry resulting from EFGM.

In this study, we conducted experiments using *Cyclosa argenteoalba* to determine whether limiting the number of female matings decreases reproductive output. We compared reproductive output among females that experienced different numbers of matings and partner males. Male spiders transfer mature sperm to their pedipalps (‘charging’) in order to inseminate females, which have two genital openings, each with spermathecae for sperm storage. In a single mating, *C. argenteoalba* males typically perform two separate courtships and palpal insertions, through which they fill each spermatheca with their sperm. In most cases, these two insertions occur within a few minutes. In this study, we define ‘typical mating’ as a mating in which a male successfully completes two palpal insertions, distinguishing it from a single palpal insertion. Accordingly, we counted a mating as half when a female received only a single palpal insertion. In this species, mutilation occurs in approximately 90% of the first typical matings for females, and mutilated females lose re-mating ability [6]. Therefore, in nature, most females mate only once with just one male during their whole lives. Nevertheless, manipulating the numbers of matings and mating partners is possible because two palpal insertions are required for successful mutilation [18]: for each palpal insertion, males are considered to damage the basal part of the scape halfway from the right or left side. After the two insertions, damage from both sides leads to successful mutilation. The manipulation of multiple matings in this study utilized this characteristic: staged mating using a male and female pair was interrupted after the first insertion (i.e. the female experienced ‘half mating’ with the first male). Subsequently, another male was coupled with the female that had undergone one palpal insertion from the first male. When the second male used the same side of the pedipalp and inseminated the same spermatheca as the first male, the scape often remained intact and the second male was able to complete the second insertion (i.e. the female experienced ‘a typical mating’ with the second male). The female thus received three palpal insertions from two different males (3PI2M group). In other words, the female experienced a higher number of matings (i.e. one and a half times) than females that completed a typical mating with their scapes mutilated. Additionally, to increase the number of mating partners while keeping the number of matings at one, another group of females (2PI2M group) was provided. In this group, females received two insertions from two half-eunuch males, with only one (right or left) intact pedipalp. In another experiment, mating was interrupted after one insertion, thus females experienced a half mating with one mating partner (1PI1M group). Females experiencing typical mating, i.e. with two insertions from one male, were used as control (2PI1M control group). Reproductive output was compared among females of the groups 3PI2M, 2PI2M and 2PI1M control and between females of groups 1PI1M and 2PI1M control. The numbers of egg sacs laid in the females’ entire lives and the numbers of eggs laid and fertilization success in each egg sac were used as indicators of reproductive output. If an increased number of matings benefits females (i.e. by raising the availability of material benefits), the reproductive output would be higher in 3PI2M than in 2PI2M and 2PI1M control females and higher in 2PI1M control than in 1PI1M females. If an increased number of mating partners benefits females, the reproductive output would be higher in 2PI2M than in 2PI1M control females.

## 2. Methods

Adult and subadult *C. argenteoalba* were collected from bamboo forests in and near Shimamoto, Osaka, Japan, from 2018 to 2020, and placed individually in a vial. During collection, we used the presence of a resting web that spiders built for their final moult as a cue to ensure that the adults were collected immediately after the final moult and that subadults were ready for the final moult. On the day of or following collection, each subadult was moulted to adult in a vial. Males were kept in a vial containing wet cotton without access to food. The females were released into the observation area (9 m × 2 m), where they built their webs and freely captured their natural prey. The observation area was separated from the natural habitat of *C. argenteoalba* by residential buildings, and non-subject spiders did not appear in the observation area. These methods ensured that none of the spiders mated before the experiment. Spiders were used at least three days after their final moult to ensure sexual maturity.

Two separate experiments were conducted, one in 2018−2019 and the other in 2020. In the first experiment, three groups of unmated females were used. For females in the first group, a male was introduced into the periphery of the female web. The male walked around the web and spun a mating thread from outside the capture area of the web to a radial thread. The male sent courtship signals by jerking the mating thread and the female responded to the signal and proceeded to the mating thread. After the first successful palpal insertion, the male was removed from the web. Subsequently, another male was introduced to the web. Approximately half of the subject females successfully received two palpal insertions from the second male. These females were then used as experimental females that received three palpal insertions from two males (3PI2M group) and were subjected to the measurement of reproductive output. One spermatheca (side unknown) was considered filled with sperm from both the first and second males, while another was filled with sperm from the second male only. Other females were unable to receive the second insertion from the second male, although they attempted it several times. Subsequent inspections revealed that the scapes of these females were mutilated. These females were not used in subsequent investigations.

For females in the second group, half-eunuch males were provided as described in Nakata [18]. After anaesthetization with CO_2_, the tip of the right or left pedipalp of each male was cut using fine scissors under the microscope. Half-eunuchs were able to use the intact pedipalp only, and by introducing two contralateral half-eunuchs into a female’s web, the females received two palpal insertions from two males (2PI2M group). Each spermatheca was filled with sperm from each male. The numbers of females that mated with right-handed males first and those that mated with left-handed males first were balanced, and the order of introduction was not biased during the experimental period. For females in the third group, a male was introduced into the web and no interventions were performed before the completion of typical mating. Thus, females received two palpal insertions from one male (2PI1M group as a control). In the second experiment, females were divided into two groups: 2PI1M and 1PI1M. For females in the latter group, the male was removed after a female received one palpal insertion and the female was not provided with any second male. The 2PI1M females were provided as described in the first experiment. In all groups, males sometimes did not begin courtship behaviour within 10 min after introduction. These males were then replaced with another male and returned to a vial. In the 3PI2M and 2PI2M groups, two pairings of a female and a male were conducted per day in most cases. Owing to limited male availability, one 2PI2M and three 3PI2M females experienced a pairing with the second male on the following day, and in addition, three 3PI2M females did so two days later. Preliminary analysis revealed that the variance in intervals between pairings of two males in the 3PI2M group did not affect reproductive output. Their copulation behaviour was recorded using a high-speed camera (Casio EXILIM EX-F1, 300FPS) and the duration of each palpal insertion was measured at a resolution of 1/300 s. Spiders were photographed after each manipulation was completed and their carapace widths were measured twice from the photo using ImageJ (https://imagej.net/ij/); the average value was used for the analysis. Owing to camera malfunctions, the duration of palpal insertions was not determined in five females. Similarly, we were unable to measure carapace width in two females. Although *C. argenteoalba* is trivoltine, only spring and summer generations were used; that is, the experimental treatments were conducted from late April to May and from July to August.

After every manipulation was completed, each female was individually introduced into an experimental cage (26 cm × 65 cm × 31 cm) lined with a transparent cling film. Five of these cages were connected to a light trap via transparent polypropylene tubes. We set up four light traps connected with five experimental cages, enabling us to maintain a maximum of 20 females simultaneously. The cages and light traps were placed in a shaded outside location. Small insects of various species were attracted to the light traps. An air current generated by an electric fan equipped with a light trap introduced prey insects into a cage where spiders freely fed on them [19]. Females constructed egg sacs on the film inside the cage and laid their eggs within them. The females were kept in the cages until they died, and the number of egg sacs laid by each female was recorded. When an egg sac was constructed, it was deliberately removed by cutting the film around it and then placed in a vial with a cotton cover. The vials were maintained at 28 ℃ until hatching occurred. Undeveloped eggs were identified based on their colour or lack of hatching after 30 d. Under a microscope, we counted the numbers of spiderlings and undeveloped eggs. Their sum was regarded as the number of eggs laid in the egg sac. A total of 514 egg sacs were collected from 85 females. Unfortunately, we found that mites had invaded the cages and were present within 53 egg sacs. Because mites may potentially feed on eggs [20], the numbers of eggs laid and spiderlings in these egg sacs were treated as missing data. For the additional five egg sacs, data were missing because of handling failures. Thus, we obtained data on the numbers of eggs and spiderlings in 456 egg sacs.

## 3. Analysis

We examined the effects of the treatments on (1) the number of egg sacs laid for each female, (2) the number of eggs laid in an egg sac, and (3) the fertilization success of each egg sac. Analysis was conducted separately for both the first and second experiments. Specifically, we first conducted multilevel regressions with a robust variance estimator, as females were nested within seasons (and additionally within years for the first experiment). As explained below, our regression models were nonlinear, included random variables and multiple covariates and contained missing values, with data not balanced across these variables. Owing to the complexity of these models, the regression coefficients could not be directly interpreted to examine the effects of treatments. Instead, we performed post-estimation by calculating the marginal predicted means of three variables representing the reproductive outputs of females using the regression results and compared these among treatment groups. All regressions and post-estimations were performed using STATA 18 (StataCorp. 2023. Stata Statistical Software: Release 18. College Station, TX: StataCorp LLC.). A detailed explanation of each regression is provided below.

(1) For the number of egg sacs, a multilevel negative binomial regression was used, with the number of egg sacs as the response variable. In this study, the mean number of egg sacs was 6.04 ± 3.32 (standard deviation (s.d.), *n* = 85), with 13 as the maximum (see §4). This figure was considerably larger than the mean of the number of egg sacs of *C. argenteoalba* that had been reported to date, that is 4.0 ± 0.8 (s.d.) [21]. The larger figure in the present study was likely owing to the artificial oversupply of prey insects, while the number of egg sacs laid in the study by Miyashita [21] was considered closer to that found in the natural situation. Therefore, we excluded data from the sixth and later egg sacs from analyses (2) and (3) (see below). This procedure reduced the mean number of egg sacs to 4.13 ± 1.46 (s.d.), which seemed comparable to the figure presented in Miyashita [21]. Additionally, data from females for which carapace width could not be measured were excluded from the analysis. The total sample size was thus reduced to 326: 156 and 170 for the first and second experiments, respectively. (2) For the number of eggs laid in an egg sac, a multilevel mixed-effects negative binomial regression was used, with the number of eggs in an egg sac as the response variable. (3) The fertilization success of each egg sac was examined in two stages during the first experiment. Owing to the absence of egg development in 15 egg sacs—a considerable number—the application of negative binomial regression was deemed inappropriate. Therefore, we first examined whether treatment affected the frequency of fertilization failure, that is, no eggs developing into spiderlings. For this purpose, we used a multilevel mixed-effects logistic regression. The response variable was set to one when no eggs developed and zero when at least one spiderling was found. Additionally, we examined the fertilization rate, defined as the ratio of developed eggs to total eggs in each egg sac, after excluding cases of fertilization failure from the data. We then conducted a multilevel mixed-effects negative binomial regression. The number of spiderlings in an egg sac was used as the response variable, and the number of eggs laid in an egg sac was included as an offset. The second experiment resulted in only two cases of fertilization failure. Therefore, we conducted only a multilevel mixed-effects negative binomial regression, with the number of spiderlings in an egg sac as a response variable and the number of eggs laid in an egg sac as an offset.

For all regressions, the treatment and carapace width of females were used as explanatory variables. The interaction between the treatment and the carapace width was not used in analysis (2) and analysis (3) for the second experiment because the use of interaction hindered the estimation of standard error for each egg sac order owing to the complexity of the regression models. For other analyses, interaction was included as another explanatory variable. The season (and year for the first experiment) was included as a random factor. For the analyses of (2) and (3), the egg sac order was included as a covariate, and interactions between the treatment and egg sac order were included in the regressions. Female ID was also used as a random factor.

To examine whether the carapace width of females and the total duration of palpal insertions received by females differed among treatment groups, we conducted a multilevel mixed-effects regression for each experiment. The treatment was used as an explanatory variable, and the season (and year for the first experiment) as random factors. Then, the marginal predicted means were compared among treatment groups. Similarly, the durations of the first and the second palpal insertions were compared separately. One female that received an unusually long palpal insertion (approximately 20 s) was excluded from the analysis of insertion duration as an outlier.

## 4. Results

**Figure 1.**
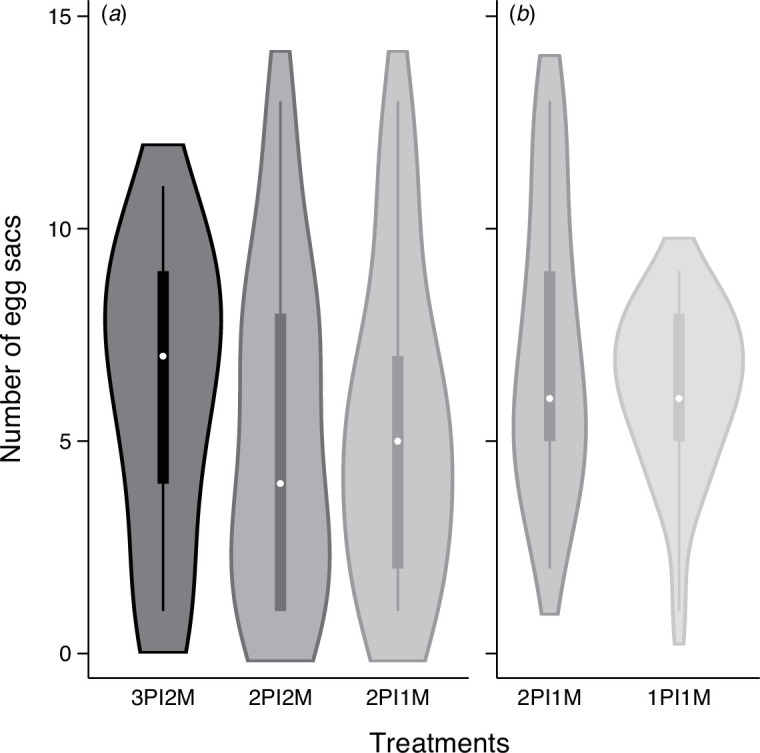
Violin plots of the total number of egg sacs laid by a female that received three palpal insertions from two males (3PI2M, *n* = 14), a female that received two insertions from two males (2PI2M, *n* = 17), a female that received two insertions from one male (2PI1M, control, *n* = 17 and 18 for the first and the second experiment, respectively) and a female that received a single insertion from one male (1PI1M, *n* = 19). (*a*) First experiment and (*b*) second experiment.

There were no significant differences in the carapace width of females among treatment groups in the first and second experiments (3PI2M: 1.49 ± 0.04, 2PI2M: 1.50 ± 0.03 and 2PI1M: 1.45 ± 0.04 in the first experiment and 2PI1M: 1.47 ± 0.04 and 1PI1M: 1.44 ± 0.03 in the second experiment, mean (mm) ± s.e.). Females that received three palpal insertions (3PI2M group, *n* = 14) laid significantly more egg sacs than those that received two insertions (2PI2M and 2PI1M groups, *n* = 17 and 17, respectively) in the first experiment (figure 1*a*, table 1; electronic supplementary material, table S1a). In other words, 3PI2M females were expected to lay 18.8−21.3% more egg sacs during their entire lifespan. In the second experiment, 2PI1M females that received two insertions (*n* = 18) laid approximately 15% more egg sacs than those that received one insertion (1PI1M group, *n* = 19); however, this difference was not significant (figure 1*b*, table 1; electronic supplementary material, table S1b).

**Table 1.** Effect of the number of partner males and palpal insertions on female reproductive output. 3PI2M: females that received three palpal insertions from two males, 2PI2M: females that received two palpal insertions from two males, 2PI1M: females that received two palpal insertions from one male (control) and 1PI1M: females that received a single palpal insertion from one male. The *p*-values underwent Bonferroni adjustment.

	number of egg-sacs	number of eggs in an egg-sac	probability of fertilization failure	number of spiderlings in an egg-sac
	marginal predicted mean ± SE	marginal predicted mean ± SE	marginal predicted mean ± SE	marginal predicted mean ± SE
first experiment				
3PI2M	6.14 ± 0.42	61.84 ± 1.54	0.051 ± 0.017	49.00 ± 3.60
2PI2M	5.06 ± 0.81	51.73 ± 3.56	0.078 ± 0.081	46.44 ± 2.65
2PI1M	5.17 ± 0.43	57.57 ± 0.68	0.120 ± 0.017	45.31 ± 0.52
				
*p*				
3PI2M–2PI2M	0.015	0.142	1.000	1.000
2PI2M–2PI1M	1.000	0.506	1.000	1.000
3PI2M–2PI1M	<0.001	<0.001	0.130	1.000
				
second experiment				
2PI1M	7.17 ± 0.48	67.25 ± 2.98		63.30 ± 2.99
1PI1M	6.24 ± 0.11	72.93 ± 1.89		63.28 ± 0.26
*p*	0.118	<0.001		0.991

**Figure 2.**
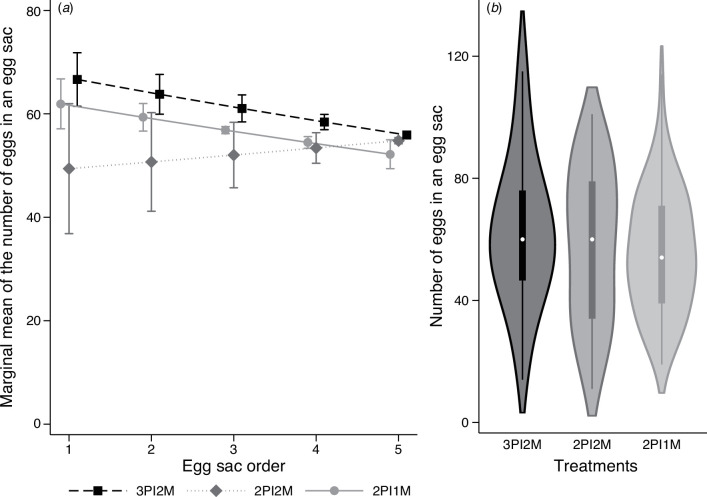
Effect of the number of matings on the number of eggs per egg sac in the first experiment. (*a*) Marginal means of the number of eggs in an egg sac along the egg sac order, estimated after multilevel mixed-effects negative binomial regression. Error bars indicate the 95% confidence interval (CI). (*b*) Violin plots displaying the number of eggs in an egg sac across three treatment groups. For descriptions of 3PI2M, 2PI2M and 2PI1M, refer to the legend of figure 1. *N* = 51, 53 and 52 for 3PI2M, 2PI2M and 2PI1M, respectively.

**Figure 3.**
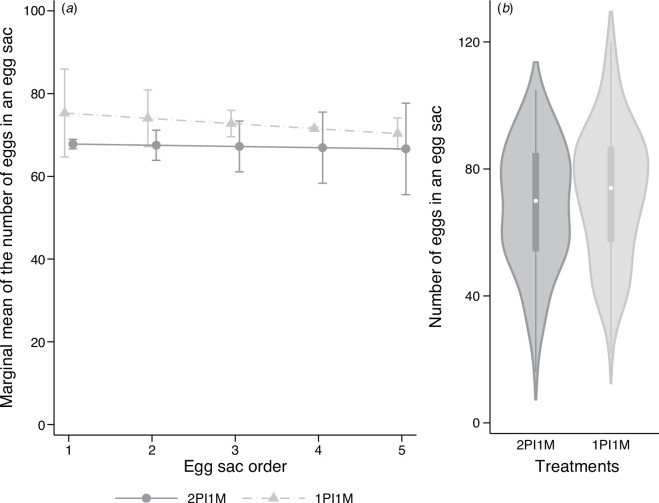
Effect of the number of matings on the number of eggs in an egg sac in the second experiment. (*a*) Marginal means of the number of eggs in an egg sac along the egg sac order. Error bars indicate the 95% CI. (*b*) Violin plots of the number of eggs in an egg sac across three treatment groups. For 2PI1M and 1PI1M, refer to the legend of figure 1. *N* = 83 and 87 for 2PI1M and 1PI1M, respectively.

Multilevel mixed-effects negative binomial regression revealed that the number of eggs laid by 2PI1M and 3PI2M females decreased as egg sac orders increased, while that of 2PI2M females did not (figure 2*a*; electronic supplementary material, table S2). The regression also indicated that the number of eggs laid increased with the carapace width of females. The marginal predicted mean of the number of eggs laid by 3PI2M females was 7.4% and 20.0% larger than that of 2PI1M and 2PI2M females, respectively (figure 2*b*). The difference in the marginal predicted means of the number of eggs between 3PI2M and 2PI1M females was significant (*z* = 5.01, *p* < 0.001, table 1). The difference between 3PI2M and 2PI2M females was not significant owing to the large standard error, although the contrast between the two groups was larger than the contrast between the 3PI2M and the 2PI1M groups. No significant difference was detected between 2PI2M and 2PI1M females (table 1). In the second experiment, the regression analysis showed a significant positive effect of carapace width on the number of eggs laid, but the effect of egg sac order was not significant (figure 3*a*; electronic supplementary material, table S3). A comparison of the marginal predicted means showed that 1PI1M females were expected to lay 8.5% more eggs than 2PI1M females, and this difference was significant (figure 3*b*, table 1).

**Figure 4.**
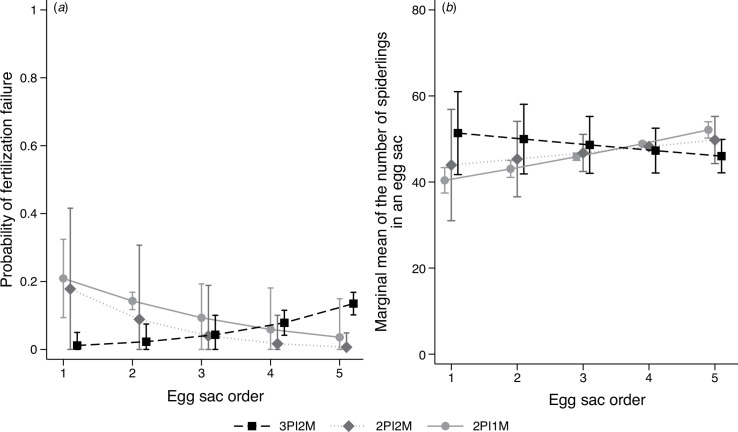
Effect of the number of matings on fertilization efficiency in the first experiment. (*a*)Marginal probability of fertilization success (at least one egg was fertilized) along the egg sac order. (*b*)Marginal means of the spiderlings in an egg sac along the egg sac order. Error bars indicate the 95% CI. For 3PI2M, 2PI2M and 2PI1M, refer to the legend of figure 1.

**Figure 5.**
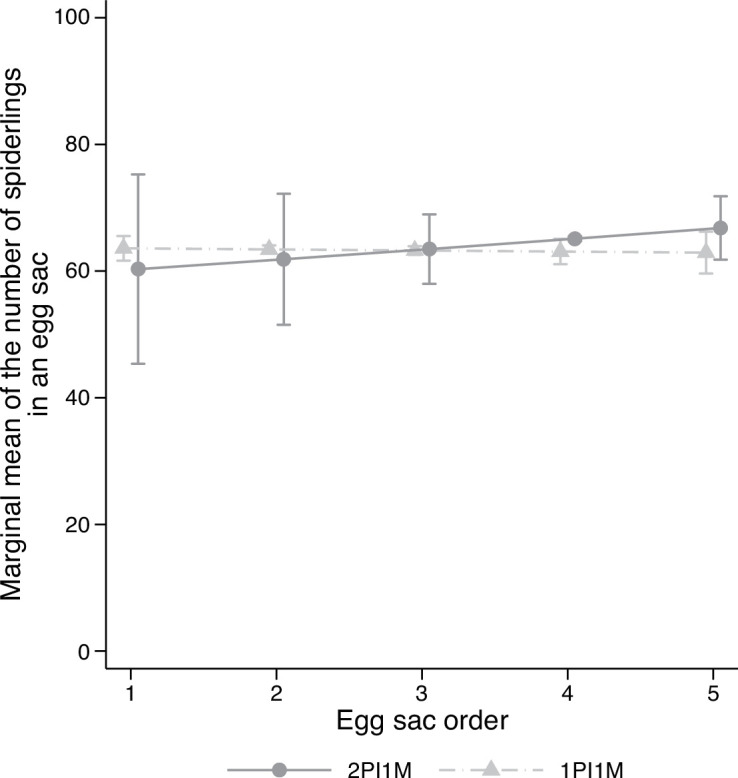
Effect of the number of matings on fertilization efficiency in the second experiment, indicated by marginal means of spiderlings in an egg sac along the egg sac order. Error bars indicate the 95% CI. For 2PI1M and 1PI1M, refer to the legend of figure 1.

Logistic regression analysis of the effect of treatment on the frequency of fertilization failure in the first experiment revealed that the fertilization failure of the 3PI2M group was initially lower than that of other groups but increased with egg sac order, while the fertilization failure of other groups decreased (figure 4*a*; electronic supplementary material, table S4a). Nevertheless, a comparison of the marginal predicted means among the treatment groups did not reveal any significant differences (table 1). The number of spiderlings in the first experiment was decreased with egg sac order in the 3PI2M group but increased in the other two groups. Nevertheless, the comparison of the marginal predicted mean did not reveal any significant difference between treatment groups (figure 4*b*; electronic supplementary material, table S4b; table 1). In the second experiment, while the regression analysis revealed the significant effect of carapace width, a comparison of the marginal predicted mean did not reveal any significant effect on the number of spiderlings (figure 5; electronic supplementary material, table S5; table 1).

The total duration of palpal insertions was, as a matter of course, significantly longer in the 3PI2M group than in the 2PI2M and 2PI1M groups for the first experiment, and significantly longer in the 2PI1M group than in the 1PI1M group for the second experiment (table 2). For the first palpal insertion, a small difference was detected between the 3PI2M and 2PI1M groups in the first experiment, i.e. the duration was only 0.05 s (=5%) longer in the 3PI2M group. There was no significant difference between the 2PI1M and 1PI1M groups in the second experiment. For the second palpal insertion, however, the duration in the 3PI2M group was more than 0.5 s, i.e. approximately 50% longer than that in the other two groups. The duration of the third insertion in the 3PI2M groups was similar to that of the first insertion.

**Table 2.** Duration of palpal insertions. For descriptions of 3PI2M, 2PI2M, 2PI1M and 1PI1M, refer to the legend of table 1. The *p-*values underwent Bonferroni adjustment.

		the duration of palpal insertion			
		total	first	second	third
	*N*	marginal predicted mean ± SE (s)	marginal predicted mean ± SE (s)	marginal predicted mean ± SE (s)	marginal predicted mean ± SE (s)
first experiment					
3PI2M	14	3.64 ± 0.13	1.06 ± 0.02	1.52 ± 0.02	1.04 ± 0.11
2PI2M	17	2.15 ± 0.10	1.14 ± 0.16	1.01 ± 0.05	
2PI1M	13	2.04 ± 0.04	1.00 ± 0.01	1.00 ± 0.08	
					
*p*					
3PI2M–2PI2M		<0.001	1.000	<0.001	
2PI2M–2PI1M		0.236	1.000	1.000	
3PI2M–2PI1M		<0.001	<0.001	<0.001	
					
second experiment					
2PI1M	19	2.12 ± 0.72	1.12 ± 0.38	0.98 ± 0.34	
1PI1M	16	1.20 ± 0.27	1.18 ± 0.27		
*p*		0.039	0.619		

## 5. Discussion

### (a) The effect of mating number on female reproductive output

The results of the first experiment indicated a positive effect of the number of palpal insertions on female egg-laying. Females that received three palpal insertions from two males (3PI2M) laid more egg sacs than those that received two palpal insertions from two males (2PI2M). They also laid more eggs per egg sac than females that received two insertions from two males, although this difference did not reach statistical significance. No significant difference was observed in the number of egg sacs or the number of eggs per egg sac between females that received two insertions from one male (2PI1M) and those that received two insertions from two males (2PI2M). These results suggest that receiving sperm from multiple males does not affect reproductive output; however, an increased number of palpal insertions does. Females that received three palpal insertions from two males (3PI2M), representing one and a half matings, laid more egg sacs and more eggs per egg sac than females that received two insertions from one male (2PI1M). This finding supports the idea that an increased number of palpal insertions benefits females, given that receiving sperm from multiple males does not influence reproductive output. Unfortunately, we could not estimate the whole-life reproductive output in this study because the data on the number of eggs were partially missing owing to predatory mites. Nevertheless, these results support the hypothesis that multiple matings benefit females.

The increased numbers of egg sacs and eggs in an egg sac of females that received three insertions imply that females suffer fitness costs from EFGM, which limits the number of insertions that females can receive to two. Three insertions increased the number of egg sacs by 18.7% and the number of eggs per egg sac by 7.4% from those of control females. Thus, a 27.5% increase in whole-life egg production was estimated. This figure may be overestimated because the prey was likely oversupplied in this study compared with natural environments. This oversupply might have exaggerated the difference in egg sac numbers between the treatment groups. In fact, the number of egg sacs laid in this study was higher than that previously reported by Miyashita [21]. However, the average number of eggs per egg sac was smaller in this study than in a previous study [21]. The artificial prey supply in this study may have biased the prey type, leading to a suboptimal nutrient balance. This may affect the physiology of the spiders [22], thereby limiting egg production. If this were the case, the effects of the treatment would have been underestimated. Nevertheless, the estimated increase in the fitness of females that received the three insertions was within the range of known fitness increases from multiple matings in insects [23].

### (b) The possible effect of genital damage and the duration of palpal insertion

The results of the second experiment were not consistent with those of the first experiment. Although females that received two palpal insertions laid more egg sacs than those that received one insertion, the difference was not significant. The number of eggs in an egg sac was larger in females that received one insertion than in control females that received two insertions; that is, the effect of the number of insertions on the number of eggs was the opposite of the result of the first experiment. However, these results should be interpreted with caution for two reasons. First, the reduction in the number of eggs per egg sac in females that receive two insertions may be compensated for by the increased number of egg sacs laid, and the whole-life reproductive output may not decrease. The former females laid 7.17 egg sacs containing 67.25 eggs and the latter females laid 6.24 egg sacs containing 72.93 eggs, on average. Therefore, their lifetime egg production was estimated at 482.2 and 455.1, respectively. Second, in *C. argenteoalba*, while approximately 90% of females lose their scapes after receiving two palpal insertions, the loss of scape is rare after one insertion. In the current study, all control females, except for one in the second experiment, were mutilated, while no females that received only one insertion were mutilated; a lower number of eggs per egg sac may indicate a direct negative effect of EFGM, likely owing to physiological stress from genital damage. Unfortunately we did not expect this possibility before the experiment, because in *L. jeskovi*, another EFGM spider species, genital damage did not appear to be related to increased physiological stress. Nevertheless, it should be noted that one unmutilated control female laid nine egg sacs, with a mean of 67.6 ± 14.9 (s.d.) eggs per egg sac, both of which were higher than the marginal predicted mean for control females (table 1). Based on the results of the second experiment, it appears premature to draw any conclusions regarding the direct negative effect of EFGM or the effect of mating frequency on female fitness. In future studies, experiments should be conducted to separate the effect of the number of insertions from that of EFGM. For example, after the artificial removal of the scape from single-insertion females, a comparison of their fecundity with that of single-insertion females with intact scapes should be considered to address this question. Physiological stress caused by genital damage can be measured directly. For example, smaller stoneflies showed lower antioxidant defence ability to oxidative stress [24]. It is possible that in *C. argenteoalba*, which is smaller than *L. jeskovi*, the loss of the scape elevates stress levels, resulting in lower fecundity.

In the first experiment, the marginal mean of the probability of fertilization success remained the same among the treatment groups. The number of hatchlings in an egg sac was also similar among treatment groups in both the first and second experiments. Given that the analysis was adjusted for the number of eggs in an egg sac, the absence of a treatment effect on the number of hatchlings indicates no treatment effect on the fertilization rate. Collectively, these results suggest that the effect of treatments on fertilization efficiency was, if present, negligible. This seems to imply that the amount of sperm from a single palpal insertion is sufficient to fertilize eggs that females lay for their whole lives, which is supported by the result of the second experiment that estimated lifetime reproductive outputs of females receiving one and two insertions to be similar. If so, the female benefits of an increased number of matings would not result from avoiding sperm depletion in *C. argenteoalba*. On the other hand, an increased number of matings resulted in a longer duration of palpal insertions in this study, which may explain the increased reproductive output in females that received three insertions. Although seminal gifts have not been detected in spiders [25], additional nutrients in the seminal fluid may also increase the egg production of *C. argenteoalba* females. Alternatively, females might utilize palpal insertion as a cue to increase egg production. A positive relationship between female fecundity and copulation duration has been established in numerous spiders [26]. In species for which the copulation duration is short, a linear relationship between copulation duration and sperm transfer was predicted [27]. Nevertheless, this relationship has not been examined in *C. argenteoalba*. A spider male can strategically alter sperm transfer in response to sperm competition [27]. In this study, the longer duration of the 3PI2M females’ second insertion, i.e. the first insertion from the second male, may indicate that the second male transfers more sperm in response to sperm competition, resulting in increased reproductive output in 3PI2M females. A second male might sense the presence of the first male’s sperm in the spermatheca during his palpal insertion. However, an alternative explanation is possible: the result may simply reflect that second males needed a longer duration to transfer their sperm into a spermatheca that is already filled with sperm from the first male, and not necessarily indicate increased sperm transfer. We assume that most 3PI2M females received the first palpal insertion of the second male by the same side of spermatheca into which the first male transfers his sperm. Otherwise, scape was removed after the first insertion of the second male and the third insertion was not possible in most cases. In any case, studying the details of transferring sperm in this species will successfully elucidate the reason for increased reproductive output in females receiving three insertions.

### (c) Sexual conflict and EFGM

EFGM has been assumed to be a male response to sexual conflict over polyandry [5,6]; however, to date, there has been no experimental support for this assumption. To the best of our knowledge, this is the first study to reveal that potential sexual conflict underlies EFGM. Being in sexual conflict, females of certain spider species may have evolved anti-mutilation traits. In *Cyclosa ginnaga*, an EFGM species closely related to *C. argenteoalba*, females are more aggressive in courting males than *C. argenteoalba* females, and the mutilation success of this species is lower than that of other EFGM species [28]. This may indicate that female aggression, not necessarily associated with sexual cannibalism, is an anti-mutilation trait. However, the rate of successful mutilation in *Cyclosa confusa* is also significantly lower than that in *C. argenteoalba*; however, females are not aggressive towards courting males [29], suggesting the presence of another anti-mutilation trait in *Cyclosa*. One possibility is the hardening of the scape to prevent mutilation by males. In several other *Cyclosa* species, such as *Cyclosa octotuberculata*, no females have been found without their scape [30]. Examining the hardness of the scapes in *Cyclosa* spiders, including non-EFGM species, would be valuable in future studies. Alternatively, females may have evolved to accept mutilation because males benefit from securing paternity and may overcome the fitness loss of females [31]. Accordingly, EFGM species may have evolved their scape to be mutilated more easily. If the scape is difficult to remove, males may forcefully tear it off and cause more severe damage to females. Unfortunately, information regarding interspecific differences in the physical properties of scapes is currently lacking.

Traumatic mating, in which the female’s genital organs are internally or externally damaged by the male partner, is observed across a wide range of animal taxa [32]. Traumatic insemination, where the male’s organ penetrates the female’s hypodermis and deposits sperm into her hemocoel [33], is a typical example of traumatic mating and functions to secure paternity [11]. EFGM is also a form of traumatic mating but is not classified as traumatic insemination. Traumatic insemination is not necessarily associated with a reduced number of female matings, and female costs often arise from the wounding itself [11]. The results of this study suggest that females of EFGM species incur reproductive costs owing to their mating opportunities being limited to one. Male strategies that restrict the number of female matings often impose costs on females. For example, female water striders *Aquarius remigis* expend more energy on locomotion when males remain on their backs as post-copulatory guards [34]. Additionally, in scorpion flies *Dicerapanorpa magna*, traumatic mating decreases the duration of subsequent mating of females, thereby increasing the paternity share of the first male, but this also reduces female fecundity [35].

Accumulating evidence on the mating behaviour of various EFGM species has revealed interspecific variability in the success rate of mutilation and its effect on limiting the number of mates per female [29]. Integrating these variabilities alongside phylogeny and the ecological characteristics of each species is crucial for elucidating the resolution of sexual conflict within each species in different ecological contexts. This involves determining whether males prevail in the conflict and become successful mutilators or if female counter-evolution renders mutilation inefficient. Thus, future studies on EFGM will provide insights into the effects of sexual conflict on the diversity of mating behaviours and the evolution of traumatic mating in animals.

## Data Availability

The datasets supporting this article have been uploaded as part of the supplementary material [36].
